# Urban–Rural Disparities in the Incidence of Diabetes-Related Complications in Taiwan: A Propensity Score Matching Analysis

**DOI:** 10.3390/jcm9093012

**Published:** 2020-09-18

**Authors:** Shu-Yu Tai, Jiun-Shiuan He, Chun-Tung Kuo, Ichiro Kawachi

**Affiliations:** 1 Department of Family Medicine, School of Medicine, College of Medicine, Kaohsiung Medical University, Kaohsiung City 807, Taiwan; 2 Department of Family Medicine, Kaohsiung Municipal Ta-Tung Hospital, Kaohsiung Medical University, Kaohsiung City 801, Taiwan; 3 Department of Family Medicine, Kaohsiung Medical University Hospital, Kaohsiung Medical University, Kaohsiung City 807, Taiwan; 4 Department of Social and Behavioral Sciences, Harvard T.H. Chan School of Public Health, Boston, MA 02115, USA; ikawachi@hsph.harvard.edu; 5 Department of Management, Kaohsiung Municipal Ta-Tung Hospital, Kaohsiung Medical University, Kaohsiung City 801, Taiwan; kmtth8079@gmail.com; 6 Institute of Health Behaviors and Community Sciences, College of Public Health, National Taiwan University, Taipei 106, Taiwan; ctkuo.tim@gmail.com; 7 School of Public Health, National Defense Medical Center, Taipei 114, Taiwan

**Keywords:** diabetes mellitus, complication, urban, rural

## Abstract

Although a disparity has been noted in the prevalence and outcome of chronic disease between rural and urban areas, studies about diabetes-related complications are lacking. The purpose of this study was to examine the association between urbanization and occurrence of diabetes-related complications using Taiwan’s nationwide diabetic mellitus database. In total, 380,474 patients with newly diagnosed type 2 diabetes between 2000 and 2008 were included and followed up until 2013 or death; after propensity score matching, 31,310 pairs were included for analysis. Occurrences of seven diabetes-related complications of interest were identified. Cox proportional hazards model was used to determine the time-to-event hazard ratio (HR) among urban, suburban and rural groups. We found that the HRs of all cardiovascular events during the five-year follow-up was 1.04 times (95% confidence interval (CI) 1.00–1.07) and 1.15 times (95% CI 1.12–1.19) higher in suburban and rural areas than in urban areas. Patients in suburban and rural areas had a greater likelihood of congestive heart failure, stroke, and end-stage renal disease than those in urban areas. Moreover, patients in rural areas had a higher likelihood of ischemic heart disease, blindness, and ulcer than those in urban areas. Our empirical findings provide evidence for potential urban–rural disparities in diabetes-related complications in Taiwan.

## 1. Introduction

Diabetes mellitus (DM) is a chronic disease that can lead to microvascular and macrovascular complications [1]. Long-term microvascular complications such as retinopathy, neuropathy, chronic kidney disease, and macrovascular complications such as cardiovascular disease (CVD), contribute greatly to the morbidity and mortality of patients with type 2 diabetes [2]. In Taiwan, the prevalence of large-vessel disease (LVD) in DM versus non-diabetic patients has been reported to be 20% and 12.9%, respectively [3]; moreover, 15.8%, 2.5%, and 1.7% of those DM patients had ischemic heart disease (IHD), stroke, and leg vessel disease, respectively [3]. Furthermore, many of these complications increase the financial liability for the government as treatment often tariffs a high medical cost, and these complications also bring physical and emotional turmoil for the patient because their frequently ends in disability; this costly clinical condition is currently one of the key drivers of economic burden [4]. In Taiwan, DM patients with complications have a higher number of outpatient visits, increased hospitalization costs, greater outpatient costs, and higher emergency room (ER) costs as compared with diabetic patients without complications [5,6]. Diabetic care is globally a critical public health issue and we should devote efforts to decrease diabetes-related complications, and therefore reduce the economic burden.

Although standard treatment guidelines for diabetes have been established, there are many factors that can impede the effectiveness of treatment and outcome [7,8]. Even if all protocols are followed precisely, factors such as sex, socioeconomic status, geographic location, and/or access to healthcare can inadvertently alter treatment outcome and cause complications [9,10]. Currently, some studies have indicated that there was a difference between rural and urban dwellers with regards to prevalence of chronic disease and outcome. A Myanmar study indicated that metabolic risk factors were more common among urban residents, whereas behavioral risk factors were higher among rural people [11]. By contrast, in United States (USA), rural populations have higher levels of chronic disease as compared with urban populations [12]; in Australia, poor health outcomes and mortality rates are higher for residents in rural areas as compared with metropolitan areas, with the worst outcomes for those living in remote areas [13]. Common chronic health conditions such as diabetes are known to illustrate these disparities. A U.S. study found that diabetic patients, in rural areas, were less likely to reach blood pressure, HbA1c targets, and renal and ophthalmic screening requirements as compared with patients in urban areas [14]. Another Australian study also demonstrated a doubling of the incidence of DM during the 15 years prior to 2005, in rural Victoria [15].

The difference between rural and urban residents can be attributed to certain social determinates. For example, rural dwellers often experience significant health disadvantages. Geographical barriers, reduced specialist and generalist services are problems of concern in rural areas. Moreover, self-management education or programs may be more popular in urban areas. All these traits may partly contribute to the divergence of DM outcomes among different residency. However, there is a lack of studies that have investigated the occurrence of diabetes-related complications associated with urbanization in Taiwan. The absence of such studies is disadvantageous for clinicians and policymakers who possibly overlook the effects their policies may have on the prevalence of DM-related complications. In addition, this ignorance could become an obstacle to good strategies for preventing DM-related complications within certain regions.

The aim of this study was to use a national sample of adult DM patients in Taiwan to determine the association between urbanization and the incidence of diabetes-related complications.

## 2. Experimental Section

Our study protocol was approved by the Institutional Review Board of the Kaohsiung Medical University Hospital (KMUHIRB-E(II)-20170151), and the approving authority waived the need for informed consent for study participation because the study was based on secondary deidentified data from the National Health Insurance Research Database (NHIRD). 

### 2.1. Data Source

This study used data retrieved between 2000 and 2008 from the Longitudinal Cohort of Diabetes Patients (LHDB), which is a sub-dataset made up of a randomly sampled cohort of deidentified patients with newly diagnosed diabetes (http://nhird.nhri.org.tw/en/Data_Subsets.html#S4). The LHDB is representative of Taiwan’s diabetic population, as it derives its samples from Taiwan’s National Health Insurance (NHI) program, which is a mandatory, single-payment system that covers more than 99% of Taiwan’s population [16]. The LHDB began in 1996 to establish a longitudinal cohort study and continued until 2013; each year approximately 120,000 random samples of newly diagnosed diabetes were recorded in Taiwan’s NHI program. To be enlisted as a diabetic patient in the LHDB, one of the following criteria must be met: (1) at least one inpatient record with the diagnosis code of diabetes or the prescription of glucose-lowering drugs, (2) at least two outpatient visits with the diagnosis code of diabetes within 1 year, or (3) one outpatient visit with the diagnosis code of diabetes, and at least one more outpatient visit with the prescription of glucose-lowering drugs within 1 year. The diagnosis code for diabetes mentioned above is also included in the ICD-9-CM (International Classification of Diseases, Ninth Revision, Clinical Modification) code 250, 648.0, or A-code A181. Thus, LHDB can be considered to be a valid national dataset, and has been referenced in numerous studies that investigated the long-term health outcomes of patients [17,18,19].

### 2.2. Study Design and Population

**Figure 1:**
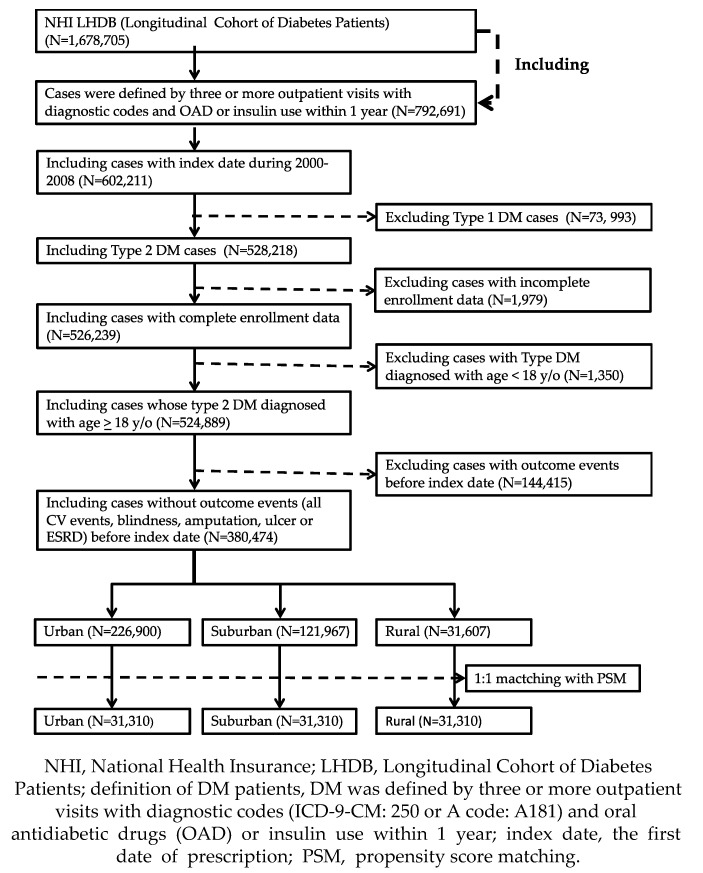
Flow chart of the screening and inclusion of study participants.

Figure 1 shows the sample enrollment procedure. First, only DM patients from LHDB with three or more outpatient visits with diagnostic codes (International Classification of Diseases, Ninth Revision, Clinical Modification, ICD-9-CM code: 250 or A code: A181) and oral antidiabetic drug (OAD) or insulin use within 1 year were selected; a total of 792,691 patients matched this criterion. Second, only patients with index date between 2000 and 2008 were included, excluding 190,480 patients. Third, 73,993 potential type 1 diabetes cases, 1979 patients with incomplete enrollment data, and 1350 patients with type 2 diabetes diagnosed when they were younger than 18 years, were eliminated from the dataset. Last, in order to estimate the incidence rates of diabetes-related complications, we further excluded all patients diagnosed with complications before the study start date, as well as patients who did not submit claims for the prescription of an OAD after the start date. In total, we identified 380,474 DM patients for study inclusion, and they were stratified into three groups based on urban, suburban, or rural residence. To reduce potential confounding, we used propensity score matching for patients living in the three types of areas using a 1:1 matching procedure based on age, insurance range, and the Charlson Comorbidity Index (CCI) score. After matching, 93,930 patients were stratified into urban, suburban, and rural groups. 

### 2.3. Variables

#### 2.3.1. Urbanization

Taiwan National Health Research Institute has stratified all 359 cities and counties in Taiwan into seven levels based on a composite score derived from population density, population ratio of people with college or higher educational levels, population ratio of older adults (age > 65 years), population ratio of agricultural workers, and number of physicians per 100,000 people (1, indicating the most urbanized, to 7, indicating the least urbanized [20]). This stratification of Taiwanese townships has been used in previous studies [21,22,23]. For analysis, we further grouped the seven levels into urban (1, 2), suburban (3–5), and rural (6, 7) areas. Figure S1 shows the mapping of the urbanization level distribution of 359 townships in Taiwan.

#### 2.3.2. Diabetes-Related Complications

Amalgamating several previous studies [6,24,25,26], we found the presence of seven DM-related complications of interest using the ICD-9-CM codes and the procedure codes. A patient was considered to have a complication if they had at least one hospitalization or two outpatient visits associated with a primary or secondary diagnosis with an ICD-9-CM code or a corresponding procedure code for that complication. Complications of interest included ischemic heart disease ((IHD) nonfatal events, ICD-9 code: 411–414.9), myocardial infarction ((MI) nonfatal myocardial infarction, ICD-9 code: 410), congestive heart failure ((CHF) ICD-9 code: 428), stroke (major stroke; ICD-9 430–434.9 or 436), blindness (ICD-9 369–369.9), end-stage renal disease ((ESRD) advanced chronic kidney disease, ICD-9 585 and 586) with hemodialysis (procedure code 39.95), or peritoneal dialysis (procedure code 54.98), and ulcer (chronic ulcer of lower limb; ICD-9 707.10). 

### 2.4. Statistical Analysis

Descriptive statistics (mean, SD, and percentage) were analyzed to examine the distribution of the characteristics of the study sample. For descriptive analysis, age was classified into <40, 40–59, and ≥60 years and insurance ranges were classified into <NT$15,000 or <US$500, NT$15,000–NT$29,999 or US$500–US$999.9, >NT$29,999 or >US$999.9; comorbidities included hypertension, hyperlipidemia, and neuropathy; medications included steroid, nonsteroidal anti-inflammatory drugs (NSAIDs), statins, anticoagulants, and diuretics; the CCI score was classified into ≤2, 3, and ≥4. Significant differences in urbanization across sociodemographic groups were evaluated using the Chi-square test. Cox proportional hazard regressions were performed to investigate the association between the level of urbanization and the occurrences of diabetes-related complications, after adjustment for the abovementioned variables. The cumulative survival estimates of all-cause mortality and all CV events between the urbanization groups were analyzed using the Kaplan–Meier method, and the difference was examined using the log-rank test. All statistical analysis was performed using the Statistical Analysis Software package, SAS version 9.1. 

## 3. Results

### 3.1. Demographic Characteristics

Table 1 presents the distributions of demographic characteristics and selected comorbidities according to the level of urbanization. There were no significant differences among the three groups with regard to age, insurance range, or CCI scores after propensity score matching. The mean age, respectively, is 56.79, 56.65, and 55.81 y/o among rural, suburban, and urban groups. The majority of included patients are male (range from 54.2 to 56.2%). Our data showed that patients with diabetes living in urban areas were more likely to have hyperlipidemia (*p* = 0.038) and use statin for treatment (*p* < 0.0001) than those living in rural or suburban areas. Moreover, patients in rural areas more frequently used steroids, NSAIDs, anticoagulants, and diuretics (all *p* < 0.0001) than those in the other two groups.

### 3.2. Cumulative Survival of All-Cause Mortality and All CV Events

**Figure 2:**
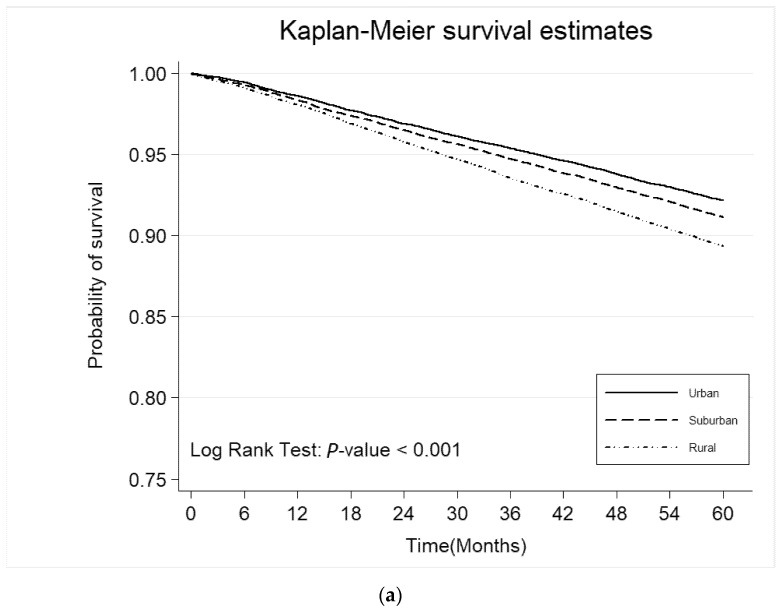

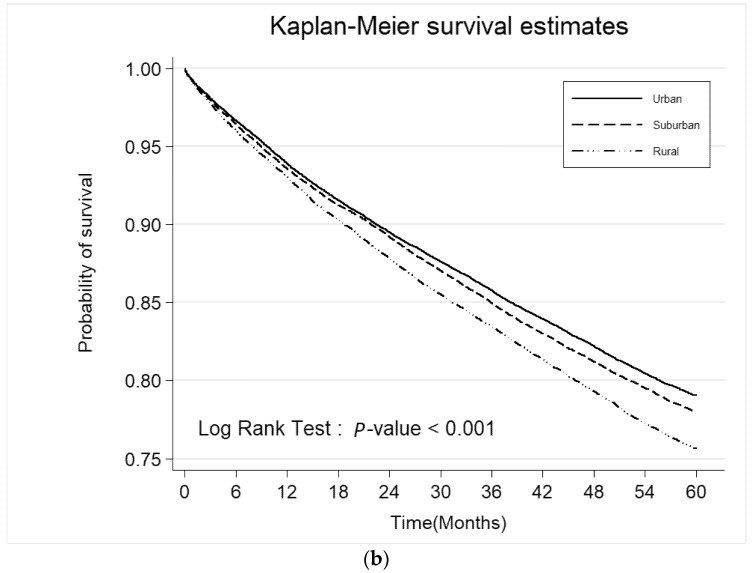
Kaplan–Meier survival curves. (**a**) All-cause mortality; (**b**) All CV events by urbanization.

Figure 2 depicts the differences in the cumulative survival rate of all-cause mortality and all CV events among three groups. During the study period, diabetic patients in rural areas had significantly higher risk to develop mortality and all CV events than those in the suburban or urban areas (Log-rank test *p* < 0.001).

### 3.3. Urbanization and Diabetes-Related Complications

Table 2 presents the details of the crude and adjusted hazard ratios (HR) for DM-related complications, by urbanization in the Cox proportional hazard regression analysis. After adjusting for the patients’ age, sex, insurance range, comorbidities, medications, and CCI scores, the HRs of all CV events during the five-year follow-up period were 1.04 and 1.15 times higher (95% confidence interval (CI) 1.00–1.07; *p* = 0.039 and 1.12–1.19; *p* < 0.001) in the suburban and rural groups, than in the urban group, respectively. Patients with diabetes in suburban areas had a greater likelihood of CHF (HR 1.09, 95% CI 1.01–1.18, *p* = 0.035), stroke (HR 1.12, 95% CI 1.05–1.19, *p* < 0.001), and ESRD (HR 1.08, 95% CI 1.00–1.17, *p* = 0.035) than the patients in urban areas. Patients with diabetes in rural areas had a greater likelihood of IHD (HR 1.09, 95% CI 1.05–1.14, *p* < 0.001), CHF (HR 1.29, 95% CI 1.19–1.39, *p* < 0.001), stroke (HR 1.24, 95% CI 1.17–1.32, *p* < 0.001), blindness (HR 2.14, 95% CI 1.20–3.83, *p* = 0.010), ulcer (HR 1.40, 95% CI 1.06–1.84, *p* = 0.016), and ESRD (HR 1.15, 95% CI 1.06–1.25, *p* = 0.001) than those living in urban areas.

## 4. Discussions

In this study, we found evidence that diabetes-related complications were more common among patients residing in rural areas than among those in urban areas. Although many countries have begun to raise awareness of factors that lead to diabetes, they have focused on exploring the relation between urbanization levels and incidence of diabetes; the outcomes have revealed that urbanization has significant impacts on the prevalence rates of diabetes in various countries [9,10,27,28,29]. However, research linking rurality and occurrence of diabetes-related complications is scarce. Although Lin et al. conducted a study that documented a higher prevalence of diabetes and stroke in areas with the highest urbanization in Taiwan, their research focused only on the link between urbanization and stroke, whereas our study investigated a more diverse set of potential factors [30]. We believe our nationwide population-based study represents the first attempt to examine the association between the urbanization and the incidence of diabetes-related complications.

A previous study indicated that the “diabetes epidemic” was growing vigorously, particularly in the urban populations of developing countries or among people who were >65 years old [27]. A systemic analysis was also conducted to explore the association between urbanization and prevalence of type 2 diabetes in southern Asia. The result found that the prevalence of type 2 DM was strongly associated with male gender, increased age, and urban residency [29]. Another study also reported higher diabetes prevalence in high socioeconomic circumstances (SEC) urban cities as compared with the low-SEC rural counties in China [9]. Despite having different study designs and definitions of diabetes or urbanization, the studies unanimously agreed that population aging and urbanization were key drivers of increasing prevalence of diabetes. The results from our study suggest that diabetes-related complications are also associated with urbanization and that improvement of the quality of diabetic care in rural areas is needed.

In this study, urbanization was defined by a composite score based on age structure, population density, immigration rate, economic activities, educational level, average family income, availability of healthcare facilities, etc. We believe that defining urbanization this way best represents the differences between levels of urbanization within each region and provides the most accurate reflection in terms of urbanization levels in different cities and counties. Figure S1 shows the geographic distribution of urbanization in Taiwan, wherein the most rural areas (Levels 6 and 7), which have been found to have the least accessibility to medical resources, are not surprisingly found located in the central mountain or eastern areas, where traveling back and forth is a strenuous expedition. This also influences the length of food supply chain that is crucial in determining the risk of developing metabolic syndrome in a population adhering to the Mediterranean diet [31]. However, we also found that some rural areas in western Taiwan had greater access to diabetes care resources since they were close to the urban areas. This finding would be a good place to begin the change we seek. Policymakers or physicians could start to understand disease management within these rural areas to develop some good strategies to prevent diabetes-related complications.

As of 2014, 99.9% of Taiwan’s population was enrolled in insurance coverage [16]. By looking at the data of NHIS, we were able to investigate how medical resources were being allocated in our country. Despite Taiwan being a small country, there is still an uneven distribution or accessibility of medical resources among rural and urban areas. This problem remains a subject of public concern, because it implies that many clinics, specialists, hospitals, and medical centers are concentrated in urban areas. More resources need to be allocated to underserved rural populations with diabetes [6]. In addition, knowledge, attitudes, and understanding of the condition can all influence patient prognosis because diabetes is a chronic disease that requires a high level of self-management [32,33]. Several studies have found that in rural areas, people were less informed about diabetes, even those diagnosed with type 2 DM [34,35,36]. Furthermore, there are differences in adherence to preventive service guidelines among rural, suburban, and urban primary care physicians because of greater distance to health care services, patient resistance to medical care, and inadequate access to resources and specialists [37]. Efforts to reduce diabetes-related complications should consider the barriers and facilitators unique to rural, suburban, and urban areas. This study also found an association, though one that was not statistically significant, between MI and urbanization among DM patients. This association is hypothesized to be caused by Taiwan’s rapid socioeconomic development, from an agrarian society to one based more upon industrial and commercial activities. Participants who live in more developed areas (with higher population density, immigration rates, and higher-speed economic activities) may experience higher levels of work stress and sedentary behavior, which, subsequently, could lead to a higher MI prevalence [38]. Similar to reports from Mexican Tarahumara, Tarahumara Indians are also susceptible to urbanization-associated worsening of cardiometabolic health, especially in men, despite their well-known and outstanding exercise performance [39]. Other studies have also reported that higher air pollution levels, often a side effect of rapid urbanization, could lead to a correspondingly higher prevalence of MI [40].

Interestingly, blindness caused by DM was more common in rural areas. One study, conducted in Hungary, indicated similar findings and attributed the higher occurrence of DM-induced blindness to lower coverage of eye screening in rural areas [41]. Patients with diabetes are recommended to undergo an annual fundus examination [7]; it is plausible that the increase of blindness in rural areas could be caused by the lack of access to healthcare. As healthcare is more accessible in urban areas and larger cities than in rural settlements, rural areas have less diabetic screening coverage and less access to eye care services [42]. Therefore, better organized primary eye care or a national screening program (e.g., telemedicine services) should be implemented, especially in rural areas, to ensure equal access to eye care services in every part of Taiwan.

This study has several limitations. First, this study lacked information on individual educational levels and marital status, both of which were likely to influence treatment outcomes and the occurrence of complications of this predominantly self-managed condition. However, previous studies have argued for a causal relationship between education and health outcome, even in diabetes [43]. Second, some unobservable confounders for individual patients (e.g., lifestyle, prescription adherence, cigarette smoking, alcohol consumption, and baseline HbA1c value) were not available. Although we matched the patients among three groups based on age, insurance range, and CCI score, caution was necessary when interpreting the association between urbanization and DM-related complications. Third, over- and underdiagnosis could have occurred because the complications of interest were defined using ICD-9-CM codes in administrative claims. Finally, in our study, the level of urbanization for each region was calculated by a composite score from several variables adopted by the government organization in Taiwan, and each was viewed as having equal contribution to urbanization (age structure, population density, immigration rate, economic activities, educational level, average family income, availability of healthcare facilities, etc.) [20]. However, it could well be that these variables have different impacts on the process of urbanization. Thus, further research should focus on exploring the relationship between the incidence of diabetes-related complications and specific dimensions, such as population density, economic activities, average family income, and availability of healthcare facilities.

## 5. Conclusions

This is the first nationwide study to examine the association between urbanization and the risk of diabetes-related complications in Taiwan. Our findings provide evidences of disparities in the incidence of diabetes-related complications between rural and urban areas in Taiwan. Future studies are required to examine the specific contributing factors associated with urban–rural differences.

## Figures and Tables

**Table 1:** Demographic characteristics of matched study cohorts by the level of urbanization.

	Urbanization						
	Rural		Suburban		Urban		*p* Value
	Cohort		Cohort		Cohort		
	(*N* = 31,310) *N*.%		(*N* = 31,310) *N*.%		(*N* = 31,310) *N*.%		
**Age**							
<40	3040	(9.7)	3018	(9.6)	3029	(9.7)	0.999
40–59	15,250	(48.7)	15,264	(48.8)	15,262	(48.7)	
≥60	13,020	(41.6)	13,028	(41.6)	13,019	(41.6)	
Mean (±SD)	56.79	(±12.7)	56.65	(±12.9)	55.81	(±12.1)	<0.001
**Gender**							
Female	14,345	(45.8)	14,338	(45.8)	13,726	(43.8)	<0.001
Male	16,965	(54.2)	16,972	(54.2)	17,584	(56.2)	
**Insurance range (USD)**							
<500	8812	(28.1)	8814	(28.2)	8802	(28.1)	1.000
500–999	20,722	(66.2)	20,722	(66.2)	20,724	(66.2)	
≥1000	1776	(5.7)	1774	(5.7)	1784	(5.7)	
**Comorbidities**							
Hypertension	9757	(31.2)	9748	(31.1)	9592	(30.6)	0.277
Hyperlipidemia	4291	(13.7)	4243	(13.6)	4454	(14.2)	0.038
Neuropathy	1043	(3.3)	1034	(3.3)	1014	(3.2)	0.802
Medication							
Steroid	16,485	(52.7)	15,871	(50.7)	15,241	(48.7)	<0.001
NSAIDs	24,302	(77.6)	23,858	(76.2)	22,842	(73.0)	<0.001
Statin	3831	(12.2)	3879	(12.4)	4467	(14.3)	<0.001
Anti-coagulants	2786	(8.9)	2564	(8.2)	2310	(7.4)	<0.001
Diuretics	13,869	(44.3)	13,248	(42.3)	11,810	(37.7)	<0.001
**Charlson’s index score**							
≤2	26,855	(85.8)	26,904	(85.9)	26,788	(85.6)	0.767
3	2575	(8.2)	2548	(8.1)	2624	(8.4)	
≥4	1880	(6.0)	1858	(5.9)	1898	(6.1)	
Mean(±SD)	1.24	(±1.4)	1.21	(±1.4)	1.23	(±1.4)	0.013

**Table 2:** The adjusted hazard ratio (aHR) of complications of diabetes mellitus (DM) patients among different levels of urbanization.

	No. Cases	Per 1000 PY	Crude HR	(95% CI)	*p* Value	aHR	(95% CI)	*p* Value
**All CV event (IHD/MI/CHF/Stroke)**								
Urban	6659	50.309	Ref.	---		Ref.	---	
Suburban	6933	52.886	1.05	(1.02–1.09)	0.005	1.04	(1.00–1.07)	0.039
Rural	7624	59.548	1.18	(1.14–1.22)	<0.001	1.15	(1.12–1.19)	<0.001
**IHD**								
Urban	4818	35.228	Ref.	---		Ref.	---	
Suburban	4818	35.380	1.00	(0.96–1.04)	0.864	0.99	(0.95–1.03)	0.584
Rural	5294	39.656	1.12	(1.08–1.17)	<0.001	1.09	(1.05–1.14)	<0.001
**MI**								
Urban	405	2.706	Ref.	---		Ref.	---	
Suburban	355	2.382	0.88	(0.76–1.02)	0.080	0.88	(0.77–1.02)	0.088
Rural	347	2.351	0.87	(0.75–1.00)	0.055	0.87	(0.75–1.00)	0.057
**CHF**								
Urban	1116	7.538	Ref.	---		Ref.	---	
Suburban	1252	8.518	1.13	(1.04–1.22)	0.003	1.09	(1.01–1.18)	0.035
Rural	1481	10.204	1.35	(1.25–1.46)	<0.001	1.29	(1.19–1.39)	<0.001
**Stroke**								
Urban	1882	12.863	Ref.	---		Ref.	---	
Suburban	2098	14.483	1.13	(1.06–1.20)	<0.001	1.12	(1.05–1.19)	<0.001
Rural	2296	16.032	1.25	(1.17–1.32)	<0.001	1.24	(1.17–1.32)	<0.001
**Blindness**								
Urban	17	0.113	Ref.	---		Ref.	---	
Suburban	24	0.160	1.42	(0.76–2.64)	0.270	1.45	(0.78–2.70)	0.244
Rural	35	0.236	2.09	(1.17–3.72)	0.013	2.14	(1.20–3.83)	0.010
**Ulcer**								
Urban	88	0.586	Ref.	---		Ref.	---	
Suburban	75	0.502	0.86	(0.63–1.17)	0.325	0.85	(0.62–1.16)	0.301
Rural	123	0.831	1.42	(1.08–1.87)	0.012	1.40	(1.06–1.84)	0.016
**ESRD**								
Urban	1107	7.467	Ref.	---		Ref.	---	
Suburban	1186	8.054	1.08	(0.99–1.17)	0.069	1.08	(1.00–1.17)	0.061
Rural	1255	8.606	1.15	(1.06–1.25)	0.001	1.15	(1.06–1.25)	0.001

aHR, multivariate Cox proportional hazards regression adjusted age, sex, insurance range, comorbidities, CCI score, and medication use; CV, cardiovascular; IHD, ischemic heart disease; MI, myocardial infarction; CHF, congestive heart failure; ESRD, end-stage renal disease.

## Supplementary Materials

The following are available online at https://www.mdpi.com/2077-0383/9/9/3012/s1, Figure S1: Urbanization of Taiwan.
