# Supplementary figures and images for: Urban–Rural Disparities in the Incidence of Diabetes-Related Complications in Taiwan: A Propensity Score Matching Analysis
Source: J Clin Med. 2020 Sep 18;9(9):3012. doi: 10.3390/jcm9093012 (PMC7565280; doi:10.3390/jcm9093012)

Urbanization levels

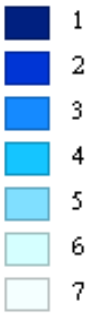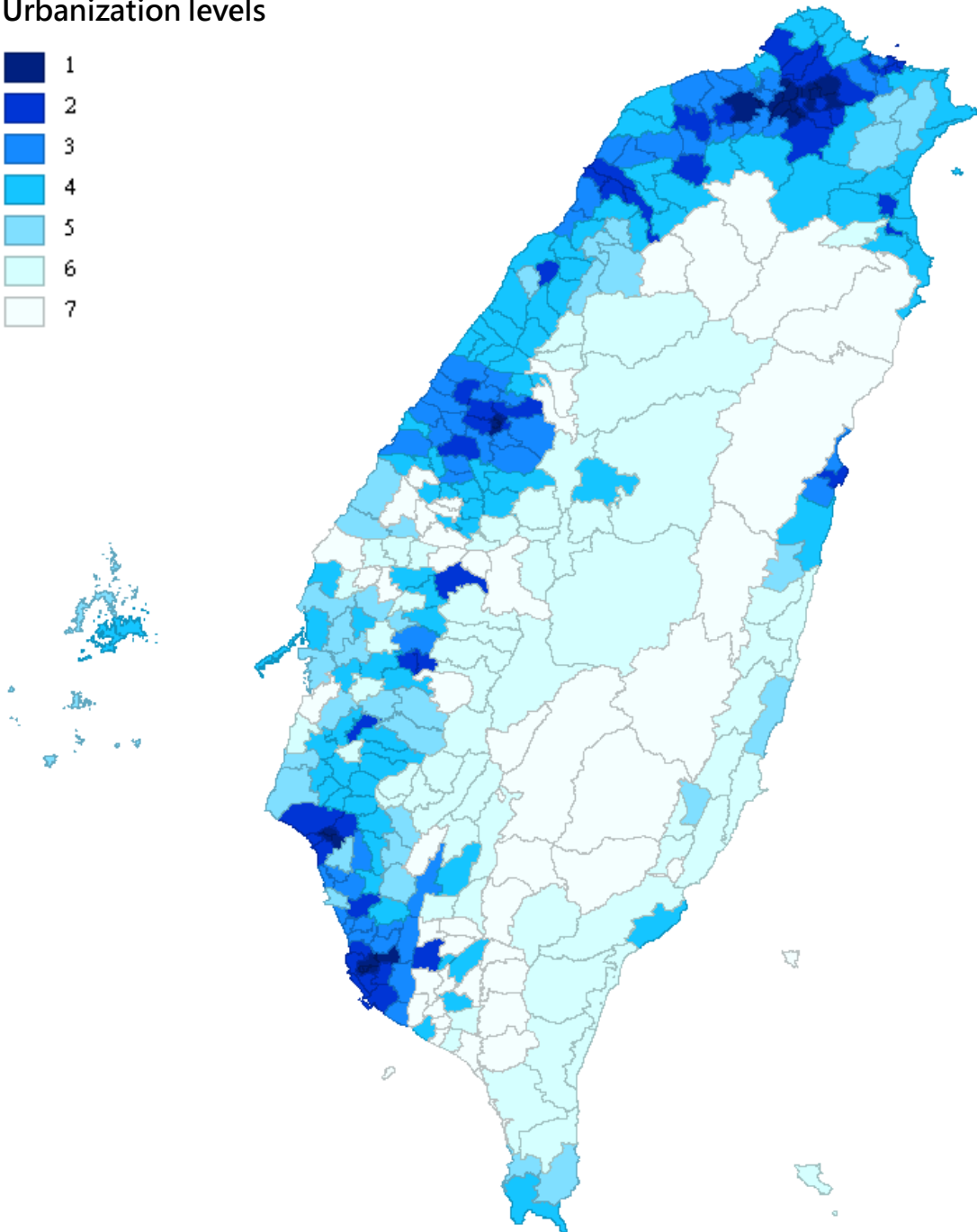

Supplement: Supplementary file 1 [file jcm-09-03012-s001.pdf]
